# Cardiovascular risk perception in women: true unawareness or risk miscalculation?

**DOI:** 10.1186/s12916-015-0351-2

**Published:** 2015-05-11

**Authors:** Miguel Cainzos-Achirica, Michael J Blaha

**Affiliations:** Department of Epidemiology and Welch Center for Prevention, Epidemiology and Clinical Research, Johns Hopkins University, Baltimore, MD USA; Ciccarone Center for the Prevention of Heart Disease, Department of Cardiology, Johns Hopkins Medical Institutions, Baltimore, MD USA

**Keywords:** Awareness, Cardiovascular disease, Risk, Risk assessment, Risk communication, Women

## Abstract

Assessing the ‘accuracy’ of cardiovascular risk perception is a worthy scientific goal that may lead to targeted interventions aimed at improving risk communication and health outcomes. Current cardiovascular risk scores, however, have shown poor calibration when used in populations that differ temporally and/or geographically from the derivation sample, limiting their reliability as the reference standard for absolute risk. In addition, accurately assessing risk awareness is challenging, with few available validated tools for effectively accounting for the outcomes assessed (coronary heart disease vs. cardiovascular disease), the time span of prediction (10-year vs. lifetime risk), and concepts of absolute versus relative risk. In this context, assessing patient awareness of the role of age as the key, non-modifiable driver of absolute risk can be particularly challenging. This commentary will examine each of these issues, providing context for the interpretation of studies on ‘discordance’ between calculated and perceived cardiovascular risk, such as the one recently published by Oertelt-Prigione et al. Moreover, we explore alternative approaches aimed at overcoming those limitations, enhancing understanding of the factors and true magnitude associated with such discordance.

Please see related article: http://www.biomedcentral.com/1741-7015/13/52.

## Background

Accurate patient perception of risk is critical for influencing behavioral change and adherence to pharmacologic treatments in the prevention of cardiovascular disease (CVD). Unfortunately, the ‘perception gap’ between calculated 10-year or lifetime risks and the risk awareness of the general population remains large [1], hindering the attainment of better health outcomes.

The phenomenon of CVD risk misperception may be particularly notable among women. CVD research and health awareness campaigns have long focused their attention on men. However, recent studies have found that women from high-income countries, for whom CVD is the number one cause of death, have strikingly low levels of awareness regarding the role of hypertension, hyperlipidemia, or tobacco use as CVD risk factors [2]. Similarly, knowledge regarding symptoms of heart diseases or their relevance as causes of female mortality has been found to be limited [2]. Importantly, focused health awareness campaigns have proved to be effective among women, with increasing levels of health education resulting in improved cardiovascular health self-care and better use of healthcare resources [3].

### Assessing risk awareness in the BEFRI Study

*BMC Medicine* has recently published a research article by Oertelt-Prigione et al. [4] showing, in a sample of 1,062 urban women from the Berlin Female Risk Evaluation (BEFRI) Study, the prevalence and independent predictors of discordance between perceived and calculated 10-year risk using the 2008 CVD Framingham Risk Score (FRS). The authors observed risk ‘discordance’ in almost 60% of the study participants, mainly as a result of subjective risk underestimation. In multivariate analyses, increasing age and some markers of low socioeconomic status were independently associated with inaccurate risk awareness.

We welcome this study, as it is among the first to address this relevant scientific question in an exclusively female population comprising diverse socioeconomic and literacy backgrounds. Furthermore, its insights may help guide targeted interventions aimed at enhancing risk communication and subsequent risk awareness among women.

### Challenges in risk assessment

**Figure 1:**
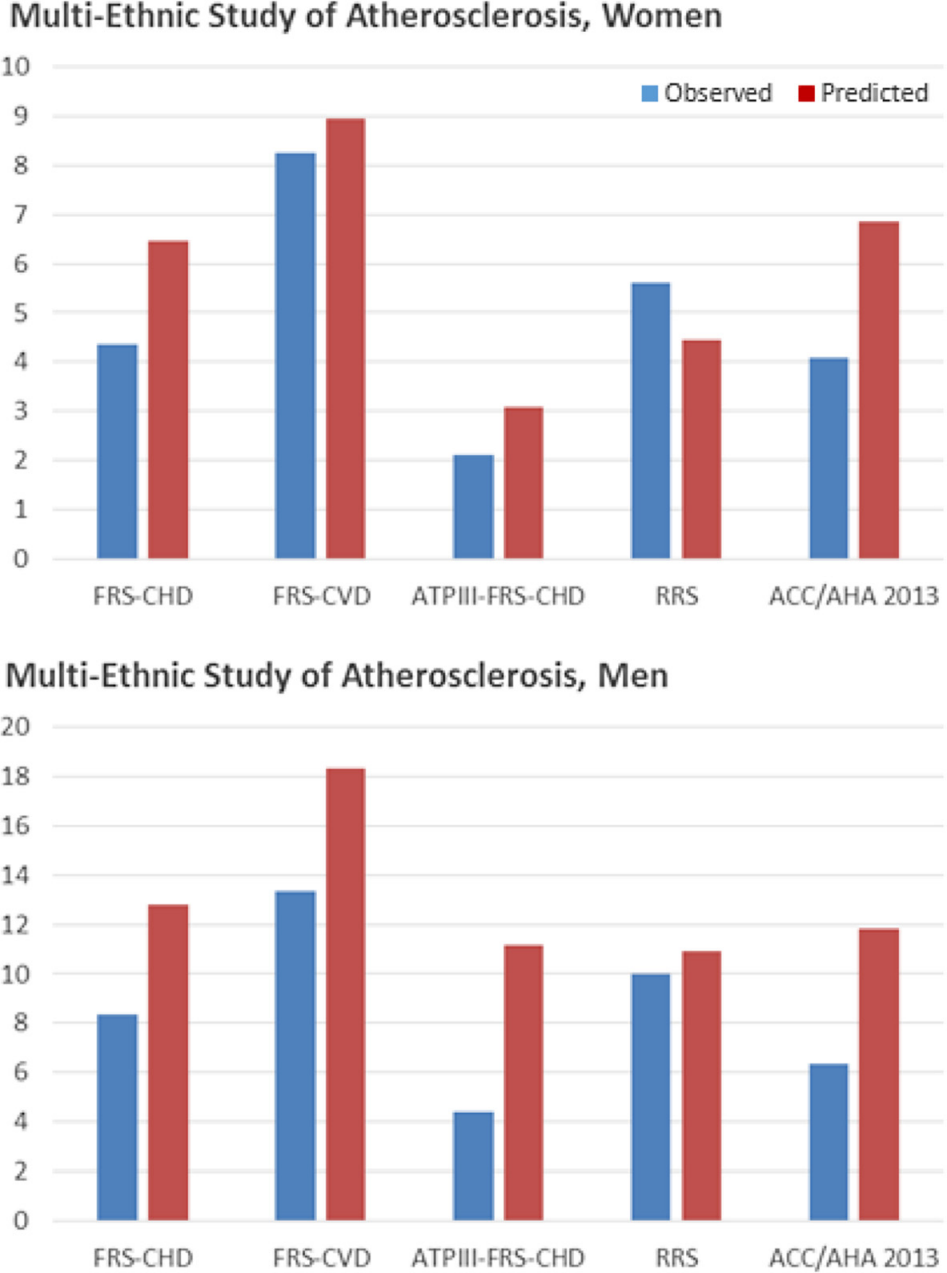
Observed versus predicted percentage of events for five US risk scores in a multi-ethnic US population – the Multi-Ethnic Study of Atherosclerosis (MESA). Reproduced with permission from the authors and adapted from [6]*.* FRS-CHD, Framingham Risk Score for coronary heart disease events; FRS-CVD, Framingham Risk Score for cardiovascular disease events; ATPIII, Adult Treatment Panel III; RRS, Reynolds Risk Score; ACC/AHA, American College of Cardiology/American Heart Association.

Accurate risk awareness, however, can only be measured after ensuring the right tools are used for the accurate assessment of both aspects of CVD risk – calculated and perceived. Regarding calculated CVD risk, even though the authors assumed score-based risk estimates as *correct* estimations, these scores at best reflect population risk (not individual risk) [5]. Furthermore, algorithms combining a limited number of single-time measured traditional risk factors have shown poor calibration when used for predicting absolute risk in modern populations. In the Multi-Ethnic Study of Atherosclerosis, four out of five widely used 10-year risk scores (including the FRS for coronary heart disease, the modified ATP-III FRS, the CVD FRS, and the ACC/AHA 2013 CVD risk estimator) overestimated risk in a multi-ethnic US population by 8% to 67% in women and by 37% to 154% in men (Figure 1) [6]. Calculated risk overestimation had also been observed in less ethnically-diverse US cohorts [7]. Importantly, the performance of scores may be particularly limited when used in populations that differ culturally, geographically, and temporally from those used for their derivation. Thus, 10-year coronary heart disease and CVD risk scores developed in the US markedly overestimate risk when used in Germany and other European countries [8,9].

### Implications of inaccurate risk assessment and alternative approaches

These limitations have important consequences. From a clinical perspective, poorly calibrated risk estimations impact the balance of anticipated benefit and harm of therapeutic recommendations, and reduce the credibility of the physician in the context of the physician-patient discussion. From a research perspective, inaccurate risk estimation hinders our understanding of the gap between perceived and actual risk. If risk discordance is observed in studies such as that by Oertelt-Prigione et al. [4], should such discordance be attributed to risk score overestimation, true unawareness, or a combination of both? We must leave open the possibility that the patient may be more accurate than the risk score. Thus, the use of the most appropriate, validated, well-calibrated risk prediction equation for the population being studied should be a priority for both clinicians and researchers. Alternatively, risk can be measured directly in prospective studies, and other powerful risk assessment tools such as atherosclerosis-imaging techniques that measure actual burden of disease can be used in cross-sectional studies for a novel way of measuring ‘risk’ discordance.

In order to best measure patients’ risk awareness, it is critical that the right questions are asked. We applaud the authors’ effort in providing the participants with background information on the risk concepts being assessed, as well as asking a broad number of questions aimed at different dimensions of CVD risk awareness. However, it is not clear whether this approach translated into actual understanding of the single 3-point Likert scale question underpinning the entire analysis. For example, the extent to which the notions of ‘low, medium, and high perceived risk’ in the general population correlate with the low, intermediate, and high FRS categories defined by the scientific community using specific 10-year risk percentage thresholds, is completely unknown. Moreover, the FRS 10-year CVD risk calculator also includes heart failure, which was not discussed with the study participants.

### The role of age and socioeconomic factors in CVD risk

The authors found that age, which is the main driver of absolute risk in most of the scores [10], was the strongest predictor of subjective risk underestimation. Understanding absolute risk can be particularly challenging, as patients may interpret risk from a relative perspective (my risk compared to that of people of similar age), excluding the role of age as a cardiovascular risk factor from the reasoning process. Further research is warranted in order to identify the best assessment tools of risk awareness, particularly in the elderly. Risk perception in younger patients is also complicated, as patients may blur concepts of short term and lifetime risk. Clearly, targeted risk communication strategies for individuals across the spectrum of age should be developed, accounting for the non-modifiable nature of age as a risk factor and explicitly tackling aspects of 10-year versus lifetime risk.

Of particular relevance are the findings regarding the role of adverse socioeconomic factors, such as joblessness, low income, or limited education, on perceived risk underestimation; such results require nuanced interpretation. Indeed, some of these socioeconomic features may result in both increased cardiovascular risk factor burden, resulting in increased absolute risk, as well as in low literacy, leading to a lack of awareness about the increased risk. In a context of economic recession recently affecting a number of European countries, the effects of social and economic stressors in both the cardiovascular health and risk awareness of the population may have important public health consequences. The 2012 European Society of Cardiology risk assessment guideline already recommends the systematic evaluation of a number of psychosocial factors as part of a standard cardiovascular risk assessment [11]. The findings from Oertelt-Prigione et al. [4] provide evidence for the further incorporation of socioeconomic factors and underscore the need for enhancing risk assessment and communication in such individuals.

## Conclusions

Research on cardiovascular risk awareness among women is still on its infancy, and the detailed characterization of this issue will require the use of the most valid, reliable tools in order to further understand the relevance and factors associated with each of the components of the process. Until then, policy-makers and healthcare providers should devote special efforts to improve risk assessment and communication in women, particularly in the elderly as well as in those facing adverse socioeconomic circumstances.

## Abbreviations

BEFRI: Berlin Female Risk Evaluation Study
CVD: Cardiovascular disease
FRS: Framingham Risk Score
